# Research on fire early warning index system of coal mine goaf based on multi-parameter fusion

**DOI:** 10.1038/s41598-023-51089-x

**Published:** 2024-01-04

**Authors:** Beifang Wang, Yuanhao Lv, Chunbao Liu

**Affiliations:** 1 School of Mines, Liaoning Technical University, Fuxin, 123000 Liaoning China; 2 Shandong Key Laboratory of Mining Disaster Prevention and Control, Shandong University of Science and Technology, Qingdao, 266590 Shandong China; 3 Bulianta Coal Mine of Shendong Coal Group Co., Ltd., Ordos, 017209 Inner Mongolia China

**Keywords:** Natural hazards, Engineering

## Abstract

In order to improve the precision of goaf fire early warning outcomes, this paper obtains the temperature characteristic index of goaf fire early warning by using a coal spontaneous combustion thermogravimetric test and a coal spontaneous combustion programmed heating test. The major gas index and auxiliary gas index for early warning are derived using the integration of the Graham coefficient and grey correlation approach. The D-S evidence theory, which involves optimizing weight allocation, is utilized to integrate the early warning temperature index and various gas indexes. Based on the fusion results, a coal mine goaf fire early warning index system is developed through multi-parameter fusion. The early warning index system is then validated through a programmed heating experiment. The results show that the process of coal spontaneous combustion can be categorized into six distinct stages: latent stage, oxidation stage, critical stage, pyrolysis stage, fission stage, and combustion stage. These stages are determined by the characteristic temperatures of coal spontaneous combustion, which are 31.7 °C, 54.8 °C, 153.7 °C, 204.5 °C, and 241.6 °C. The major gas index for early warning of goaf fires can be determined by 100∆(CO)/∆O_2_(%). Additionally, auxiliary gas indexes such as C_3_H_8_/CH_4_, C_3_H_8_/C_2_H_6_, C_2_H_4_/C_2_H_6_, and C_2_H_2_ can be used for goaf fire early warning. The programmed heating experiment shows that the early warning system software is designed by the multi-parameter fusion goaf fire early warning index system is accurate and effective. The selection of the goaf fire early warning index is more rational and precise when using the multi-parameter fusion goaf fire early warning index system based on the D-S evidence theory of weight allocation. It offers robust support for enhancing the goaf fire early warning index system and predicting coal mine goaf fires.

## Introduction

The coal resource is a crucial foundation of national economic development, and it has accounted for approximately 55% of China's energy structure in recent years. Despite efforts to promote the energy revolution and adjust the energy structure, the dominant role of coal in the energy sector remains unchanged in the near term. Moreover, the subsequent expansion of coal mining, characterized by large-scale operations and high intensity, has resulted in a number of production safety issues^1–5^. In the frequent coal mine disasters in China, coal mine fires consistently contribute to a significant fraction of these incidents^6^. The monitoring and integration of coal temperature and the coal spontaneous combustion gas index have considerable importance in achieving early warning for coal mine goaf fires.

At present, scholars at home and abroad have done a lot of research on goaf fire early warning. K.aris et al.^7^ monitored the formation rates of CO and CO_2_ at different temperature stages, and obtained the gas characteristics of coal spontaneous combustion by low temperature oxidation. Karolina et al.^8^ discussed the adsorption and resolution of gas by coal at different temperatures, and found that compared with C_2_H_4_ and other gases, the analytical range of C_2_H_6_ and C_3_H_8_ is larger. Levi et al.^9^ developed a laboratory gas evolution test to assist with identifying the most relevant gases and gas ratios for trending, it is concluded that the concentration of CO increases with the increase of temperature, and C_2_H_4_ only appears at high temperature. Sahu et al.^10^ studied 30 coal samples from 7 different coalfields in India and measured their initial temperature. The results show that the increase in temperature is a sign of spontaneous combustion tendency. Qu et al.^11^ tested the programmed temperature characteristics and infrared functional groups, analyzed the critical temperature of the coalification process, and established a spontaneous combustion prediction method based on the critical temperature variation characteristics. Yu et al.^12^ determined the activation energy and temperature of the spontaneous combustion stage of three kinds of coal by programmed heating experiment, thermogravimetric differential scanning calorimetry, and a theoretical analysis model and divided the low-temperature oxidation of coal into four stages with a specific temperature range. Gürdal et al.^13^ studied the spontaneous combustion of coal in the Canakkale basin and determined the composition of gases produced during coal combustion. Wen et al.^14^ established a model for calculating the concentration of CO in the return air corner according to the mechanism, source, and influencing factors of CO. Ma et al.^15^ obtained the effect of different concentrations of methane on coal spontaneous combustion through a low-temperature oxidation experiment and an infrared spectrum experiment on coal, combined with Pearson correlation analysis. Si Junhong^16^ established a gas-coal-fire coupled disaster ethical risk assessment model based on the Analytic Hierarchy Process (AHP) by analyzing the main control factors affecting the coupling disaster of gas and coal spontaneous combustion in goaf. Deng et al.^17^ established the XK-VI coal spontaneous combustion experimental system and obtained the spatio-temporal dynamics of high temperature zone migration and gas concentration variation during coal spontaneous combustion. Zheng et al.^18^ found that, based on the specially designed thermal experimental platform and numerical simulation method, the results show that the ventilation air leakage in the goaf is positively correlated with the spontaneous combustion intensity of coal and negatively correlated with the gas accumulation range in the goaf. When the air leakage can support the high-temperature combustion of coal in the goaf, the distribution range of O_2_ concentration is basically unchanged. Wei et al.^19^ proposed, based on the determination of the spontaneous combustion tendency of ventilated coal seams, thermogravimetric analysis and differential scanning calorimetry analysis of coal samples, optimization of gas spontaneous combustion index and division of spontaneous combustion into "three zones" with O_2_ concentration as index, pressure balance method, and ground pressure sealing method are proposed to prevent spontaneous combustion in goaf. Wang et al.^20^ studied the relationship between O_2_ consumption and emissions of various gas products and made a multiple linear regression analysis of O_2_ consumption rate and gas products. He also proposed the ratio of O_2_ consumption rate to gas product emission rate as an evaluation index to judge the state of spontaneous combustion. Yan et al.^21^ preliminarily determined the reaction intensity of spontaneous combustion and oxidation of coal with different O_2_ concentrations and different kinds of coal by studying the mathematical variation of O_2_ consumption rate with temperature and O_2_ concentration. Lu^22^ obtained the critical O_2_ concentration of coal spontaneous combustion and the predicted temperature range of different gases by comparing and analyzing the laws of gas products, the change in coal temperature, and the change in O_2_ consumption rate. Guo et al.^23^ determined the relationship between index gas concentration and coal temperature by using the Logistic function, divided coal spontaneous combustion into seven stages, and established an early warning system of coal seam spontaneous combustion including six indexes. Pone et al.^24^ studied the spontaneous combustion of coal seams in Witbank and Sasolburg coalfields in South Africa and detected high concentrations of CO, CO_2_, and CH_4_. It was found that the concentration of these gases was closely related to the temperature of coal spontaneous combustion. Xiao et al.^25^ measured seven characteristic temperatures of coal samples in the Yanzhou mining area by thermogravimetric analysis and a 15Mt super-large spontaneous combustion experimental platform, detected a specific temperature range, and established the relationship between exponential gas and characteristic temperature. Niu et al.^26^ carried out experimental determination of coal samples in Anyuan Coal Mine, and the data measured in the laboratory were initialized to "K" value. It was found that there was a high correlation between the ratio of gas to K value, such as CO, C_2_H_6_, C_2_H_2_, and coal spontaneous combustion in different temperature ranges. Wang et al.^27^ determined the gas indexes and critical values of different oxidation stages by studying the formation of CO, C_2_H_4_, C_2_H_6_, and other gases with different particle sizes in the oxidation process and put forward a three-level early warning index system for coal spontaneous combustion. Jiang^28^ combined with grey relational analysis, selected five indexes suitable for predicting coal spontaneous combustion in five different temperature stages and constructed a coal spontaneous combustion prediction system, which is mainly based on C_2_H_6_, C_2_H_6_/C_3_H_8_ and C_3_H_8_ prediction indexes and supplemented by the last three prediction indexes. Zhao et al.^29^ based on tunable semiconductor laser absorption spectroscopy and multiplexing phase locking technology, designed a set of highly integrated real-time on-line analysis and monitoring systems for multi-index gases. Ren^30^ used a logistic function to fit the change curve of marker gases, constructed a coal spontaneous combustion early warning system based on gas statistical characteristics, and divided the dangerous stages of coal spontaneous combustion. Kong et al.^31^ carried out fitting analysis of the variation curve of index gas data with coal temperature by combining logistic fitting model, and determined CO, O_2_, φ (CO)/φ (O_2_), C_2_H_4_, C_2_H_6_, φ(C_2_H_4_)/φ(C_2_H_6_) as the index gases for predicting and forecasting CSC. Tan et al.^32^ analyzed the correlation characteristics of coal spontaneous combustion related indexes by using programmed heating tests and the grey relational analysis, refined the coal spontaneous combustion early warning mechanism, and put forward a complete set of goaf fire early warning analysis processes.

Recently, both domestic and international studies primarily utilize unilateral early warning indicators, such as the characteristic temperature of spontaneous combustion or gas data from coal samples, to establish an early warning system. However, the predicted data often deviates significantly from the actual monitoring results, resulting in inaccurate warnings for goaf fires. This research categorizes the entire process of coal spontaneous combustion into six distinct stages. The test findings and Graham coefficient are utilized to examine the major early warning gas indexes. The grey correlation approach is then employed to choose the auxiliary gas indexes for early warning. Subsequently, the coal temperature and various types of early warning gas indicators are enhanced and combined using the D-S evidence theory, which is based on weight allocation optimization. This process leads to the development of a goaf fire early warning index system that utilizes multi-parameter fusion. The reliability of the early warning index system is confirmed through a programmed heating test. This facilitates the development of a more methodical and precise goaf fire early warning index system, enhances the precision of coal mine goaf fire forecasting, and guarantees the safe and efficient operation of the mine.

## Study area

Lijiahao Coal Mine of Shenhua Baotou Energy Co., Ltd. is located in the central and southern part of the Dongsheng mining area in Ordos, which is a typical mining mine with shallow depth and a short-distance thick coal seam. Nowadays, coal seams of 2-2 have been extracted, while coal seams of 3-1 are now undergoing extraction. The resource conditions and mining conditions of Lijiahao Coal Mine are as follows: the coal seams known as 2-2 and 3-1 are mostly composed of long flame coal and non-stick coal. These coal types exhibit modest levels of metamorphism and are characterised by the presence of pyrite nodules or thin films. Furthermore, they are found to occur in a stable manner and possess considerable thickness. The geological structure and hydrological circumstances exhibit a reasonably straightforward spontaneous combustion, hence creating a conducive environment for the occurrence of coal spontaneous combustion; The process of large-scale and high-intensity coal mining results in the fragmentation of residual coal in the goaf, hence creating favourable conditions for spontaneous combustion of coal; The coal seam is situated at a shallow depth and in close proximity, facilitating the formation of a compound goaf following coal extraction. The presence of mining cracks in the overlaying rock facilitates a direct connection to the surface atmosphere, hence creating favourable conditions for the spontaneous burning of remaining coal in the goaf.

Therefore, the presence of residual coal in the goaf of Lijiahao Coal Mine presents favourable conditions for spontaneous combustion, thereby posing a concealed safety hazard. The implementation of a multi-parameter fusion-based early warning index system for goaf fires can enhance the monitoring and analysis of fire prevention and control in the goaf of Lijiahao Coal Mine. This development holds significant importance for ensuring the safe and efficient production of the coal mine.

## Experimental and methods

To determine the early warning index of goaf fire, coal samples from the 2-2 and 3-1 coal seams were chosen for conducting a thermogravimetric test and a programmed heating test to assess coal spontaneous combustion.

**Figure 1:**
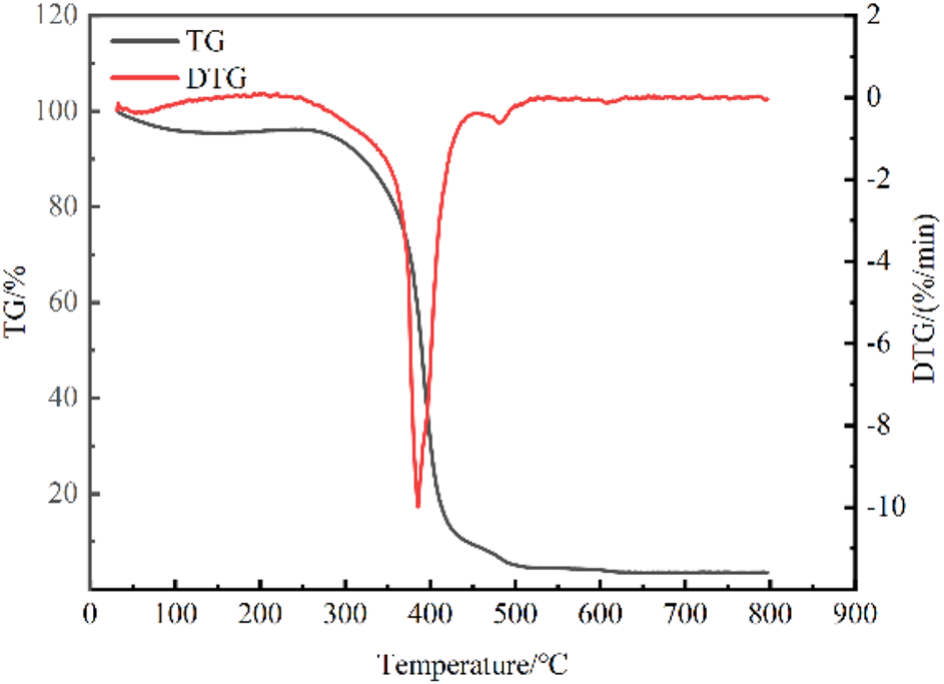
TG-DTG curve of coal samples.

Thermogravimetric-mass spectrometry (TGA-MS) was employed to analyse the mixed coal samples derived from two distinct coal seams. The experimental parameters are outlined as follows: the rate of heating is 5 °C per minute, while the flow rates of oxygen and nitrogen are 10 mL per minute and 40 mL per minute, respectively. The mass of the sample is 10 mg, and the temperature range for the reaction is between 20 and 800 °C. The TG-DTG results obtained by thermogravimetric analysis of mixed coal samples are shown in Fig. 1.

Based on the coal combustion thermogravimetric analysis test, the process of coal spontaneous combustion can be categorised into six distinct stages: latent stage, oxidation stage, critical stage, pyrolysis stage, fission stage, and combustion stage. These stages are derived from the underlying "three-stage" framework of coal spontaneous combustion. The temperature ranges are as follows: below 31.7 °C, 31.7–54.8 °C, 54.8–153.7 °C, 153.7–204.5 °C, 204.5–241.6 °C and above 241.6 °C.

The programmed heating test system was employed for the analysis of mixed coal samples. The experimental parameters are outlined as follows: The particle size ranges from 180 to 380 μm, with a weight of 0.4–0.8 g. The gas supply flow is set at 100 ml/min with an O_2_ concentration of 21%. The test furnace temperature is maintained at 25 °C. The heating rate is programmed to increase at a rate of 1 °C/min from 25 to 80 °C, 1.5 °C/min from 80 to 200 °C, and 2.0 °C/min from 200 to 300 °C. The variation data of various gas products with coal temperature in the process of coal spontaneous combustion are shown in Table 1.

**Table 1:** Test data on the concentration of oxidation gas products in coal samples.

Temperature/°C	CO/ppm	CO_2_/ppm	CH_4_/ppm	C_2_H_4_/ppm	C_2_H_6_/ppm	C_2_H_2_/ppm	C_3_H_8_/ppm	O_2/_%
25.00	0.93	15.73	10.53	0.00	0.00	0.00	0.00	20.90
35.00	1.26	35.59	11.68	0.00	0.00	0.00	0.00	20.84
45.00	2.50	47.17	11.81	0.00	0.00	0.00	0.00	20.65
55.00	6.35	52.62	11.85	0.00	0.14	0.00	0.00	20.81
65.00	5.91	82.35	12.10	0.00	0.22	0.00	0.00	20.72
75.00	37.50	162.65	12.25	0.00	0.24	0.00	0.00	20.65
85.00	80.04	308.70	12.94	0.00	0.26	0.00	0.12	20.47
100.00	150.95	536.40	14.16	0.33	0.67	0.00	0.16	20.40
125.00	1067.36	2980.51	18.36	0.67	0.39	0.00	2.40	20.18
153.80	1967.62	4614.14	41.05	3.80	12.76	0.00	10.01	17.24
205.70	4242.28	8160.43	146.51	33.59	51.71	0.00	73.98	14.14
248.50	17,053.41	36,671.53	262.14	108.49	62.43	0.98	159.03	11.35
287.40	29,243.40	64,361.02	487.48	201.16	85.32	2.23	267.52	8.07
300.20	31,411.00	74,043.00	673.09	235.06	124.16	5.76	323.80	7.04
320.10	37,069.00	91,512.00	1348.04	310.55	310.55	21.74	397.30	6.56
332.00	40,127.00	101,142.12	2083.12	352.95	352.95	40.23	418.07	6.05

According to the data presented in Table 1, there is an exponential increase in the concentrations of CO and CO_2_ in a single gas as the temperature of coal increases. Furthermore, these two gases exhibit a strong positive correlation with each other. On the other hand, gases such as C_2_H_6_, C_2_H_4_, and C_3_H_8_ also have a positive correlation with temperature, but this correlation becomes significant only after reaching a temperature of 100 °C. Additionally, the concentrations of these gases likewise the concentration of O_2_ involved in the process exhibits a reduction as the temperature increases, and experiences a rapid decrease at a critical temperature threshold.

## Discussion

Although there is a significant correlation between CO and other gases and temperature during coal spontaneous combustion, it is crucial to acknowledge that this process is non-linear. Furthermore, the goaf area exhibits sudden alterations and intricate environmental variables. An examination of the perilous state of coal spontaneous combustion, relying exclusively on a solitary gas indicator, is vulnerable to the impact of external factors. This study seeks to overcome the constraints of single gas index early warning analysis by suggesting the utilization of a compound gas index for the early detection of coal mine goaf fires.

### Selection of early warning gas index

#### Selection of major gas indicators

The analysis of the programmed heating test results for mixed coal samples revealed that the gas indexes prior to reaching 90 °C consist solely of CO, CO_2_, and CH_4_. However, coal samples demonstrated a significant ability to adsorb CO_2_ and CH_4_, with the adsorption characteristics changing depending on prevailing conditions. Simultaneously, there were multiple sources of underground CO_2_, and their concentration was affected by various factors. CH_4_, C_2_H_6_, C_2_H_4_, C_3_H_8_, and C_2_H_2_ were specifically detected in some areas during the phenomenon of coal spontaneous combustion. In contrast, CO and O_2_ have a smaller impact and are actively involved in every stage of coal spontaneous combustion. The coal spontaneous combustion's primary compound gas index was determined as 100Δ(CO)/ΔO_2_ (%), based on the previous analysis and in conjunction with the Graham coefficient.

However, it is essential to consider the complex conditions found in the coal mine goaf and the unequal distribution of oxygen levels when evaluating the oxygen concentration of the compound gas index. Therefore, it is crucial to analyze the changes in the ratio of 100Δ(CO)/ΔO_2_ (%) in mixed coal samples at different temperatures, taking into account the initial O_2_ concentration. The experiment involved doing a programmed heating test on a mixture of coal samples comprising 2-2 coal and 3-1 coal. The test was conducted using initial O_2_ volume fractions of 21%, 10%, and 7%.

**Figure 2:**
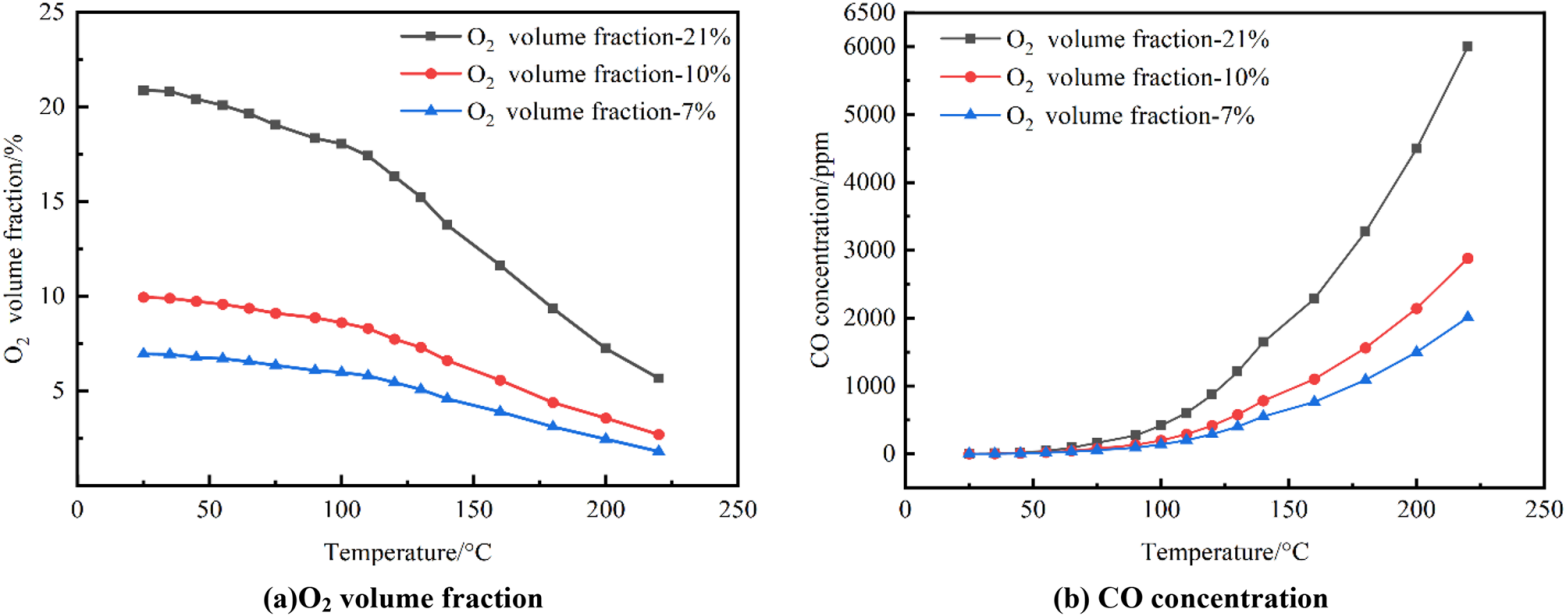
The change curves of O_2_ and CO.

The O_2_ volume fraction and CO concentration change curves in the furnace were derived from a controlled heating experiment utilizing a mixture of coal samples with O_2_ volume fractions of 21%, 10%, and 7%, as shown in Fig. 2.

According to Fig. 2, it is evident that as the temperature increased, there was a gradual decrease in the proportion of O_2_. The fall progressed steadily and consistently until it reached a temperature of 100 °C, at which point it experienced a sudden and significant increase in speed. The resulting change curve had a similarity to a parabola thrown horizontally. CO will be generated throughout the entire process. The concentration of CO increases with greater temperatures, and the overall change pattern resembles an exponential function. The rise in O_2_ concentration at identical temperatures resulted in a corresponding elevation in volume fraction as well as an increase in CO concentration.

**Figure 3:**
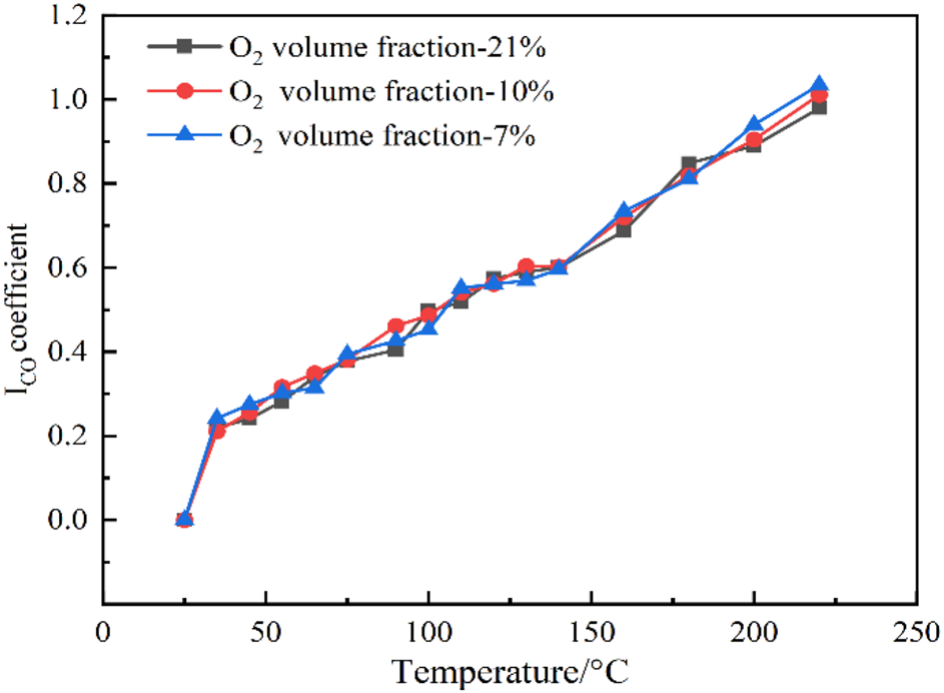
I_CO_ coefficient of each oxygen concentration.

The investigation clearly demonstrated that a higher oxygen concentration resulted in a faster decrease in the proportion of oxygen in the furnace. This suggests an elevated level of oxygen utilization. However, a noticeable pattern could be observed in the correlation between the concentration of CO and the ratio of the two variables. By combining the Graham coefficient with 100(CO)/O_2_(%), one can calculate the compound gas index of fire early warning in coal mine goaf. The experimental data demonstrates a correlation between the Graham coefficient ICO and mixed coal samples at different oxygen concentrations, as shown in Fig. 3.

Figure 3 clearly demonstrates that when the temperature increased, there was a progressive rise in the I_CO_ coefficient of coal samples. The increasing trend exhibited a high degree of stability, with the change curve aligning closely with this trend. At different levels of O_2_ concentration, the change in the I_CO_ coefficient of the coal sample was small, and the alteration pattern closely matched. This finding confirmed that the I_CO_ coefficient is a reliable gas indicator for early fire warning in coal mine goaf.

#### Selection of auxiliary gas index

**Figure 4:**
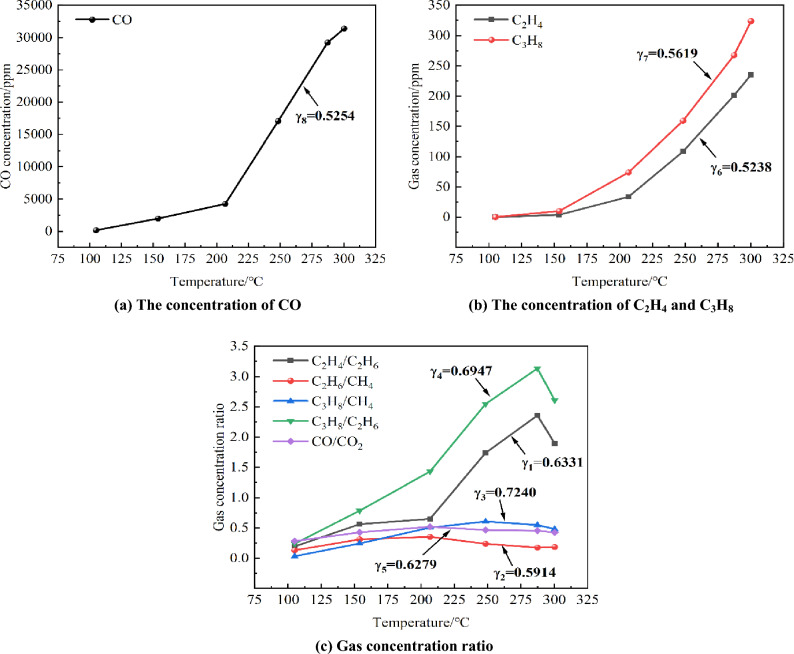
The changes of auxiliary gas indexes.

Within the local temperature range, there is a significant correlation between C_2_H_4_ and other gases and the coal temperature. Therefore, for quantitative analysis, we select the concentration of oxidation products in coal samples at the critical temperature range of 100 °C to 315 °C. Additionally, the auxiliary compound gas index for early warning of coal spontaneous combustion is determined based on the operational outcomes within the same temperature range. Figure 4 displays the temperature and auxiliary gas indicators along with their corresponding concentration ratios.

Made the temperature as the reference series, C_2_H_4_/C_2_H_6_, C_2_H_6_/CH_4_, C_3_H_8_/CH_4_ and so on as the comparison series $x_{1} \sim x_{8}$, and calculate the correlation degree.

1 $$\gamma_{i} = \gamma_{i} \left( {x_{0} ,x_{i} } \right) = \frac{1}{6}\sum\limits_{k = 1}^{6} {\varepsilon_{i} \left( k \right)}$$

In the formula: $\gamma_{i}$ is the correlation degree, $\Delta_{i} (k)$ is the absolute difference, $\varepsilon_{i} (k)$ is the correlation coefficient, and $\rho$ is the resolution coefficient, with a value of 0.5, ${\text{minmin}}\left| {x_{0} (k) - x_{i} (k)} \right| = 0.0060$ ${\text{minmin}}|{{\text{x}}}_{0}({\text{k}})-{{\text{x}}}_{{\text{i}}}({\text{k}})|=0.0060$,${\text{maxmax}}\left| {x_{0} (k) - x_{i} (k)} \right| = 1.0332$.

It was calculated that $\gamma_{1} = 0.6331$, $\gamma_{2} = 0.5914$, $\gamma_{3} = 0.7240$, $\gamma_{4} = 0.6947$, $\gamma_{5} = 0.6279$,$\gamma_{6} = 0.5238$, $\gamma_{7} = 0.5619$, $\gamma_{8} = 0.5254$.

By comparing the size of the obtained,$\gamma_{1} \sim \gamma_{8}$, $\gamma_{3} > \gamma_{4} > \gamma_{1} > \gamma_{5} > \gamma_{2} > \gamma_{7} > \gamma_{8} > \gamma_{6}$ could be obtained, and C_3_H_8_/CH_4_, C_3_H_8_/C_2_H_6_, C_2_H_4_/C_2_H_6_ was selected as the auxiliary gas index. Therefore, C_3_H_8_/CH_4_, C_3_H_8_/C_2_H_6_, C_2_H_4_/C_2_H_6_ and C_2_H_2_ were regarded as auxiliary gas indexes for early warning of coal spontaneous combustion in the temperature range of 100 °C to 315 °C.

### Multi-parameter fusion of early warning indicators

The D-S evidence theory was used to create a mathematical model of the gas index and temperature of coal spontaneous combustion. The early warning index parameters of coal spontaneous combustion were then looked at from different angles. The accuracy of the goaf fire early warning index method is higher, resulting in more precise early warning outcomes. This paper presents an analysis of many parameters, including temperature, the ratio of 100Δ (CO)/ΔO_2_ (%), C_3_H_8_/CH_4_, C_3_H_8_/C_2_H_6_, C_2_H_4_/C_2_H_6_ and C_2_H_2_.These parameters were utilised as supporting evidence within the framework of D-S evidence theory, leading to the acquisition of novel findings. The best fusion calculation of D-S was performed based on weight allocation in order to prevent the occurrence of synthesis findings that were inconsistent with common sense due to significant conflicts between the evidence. The acquisition of a scientifically sound and dependable goaf fire early warning index system were achieved.

1. D-S evidence theory
  The D-S evidence theory is well-suited for addressing challenges characterised by uncertainty. The integration of characteristic factors related to the early warning of coal spontaneous combustion allows for the determination of the credibility of the coal spontaneous combustion early warning system. The advantages of this approach encompass a robust theoretical foundation that effectively addresses randomness and fuzzy uncertainty. It achieves this by employing a trust function to quantify the level of support, even in the absence of precise probabilities. Additionally, it steadily narrows down the set of hypotheses over time. The fundamental concept involves establishing a correlation between all available evidence and a recognition framework. Subsequently, a basic probability function is generated, which is then integrated with fresh evidence using the D-S rule in order to arrive at a choice. All the early warning parameters in this paper can be regarded as different pieces of evidence.
2. Fusion process
  ① Let the possibility of fire occur as the existing decision problem $X$, the set of all possible results of the problem was set $\Omega$ (occurrence, uncertainty, non-occurrence), all the elements in the set $\Omega$ were mutually exclusive, and any proposition corresponds to a subset of $\Omega$. $\Omega$ was called the identification framework of D-S evidence theory.
    **Definition 1**
    Basic probability assignment function ($BPA$):
    **Definition 2**
    Trust function ($$Bel$$)
    **Definition 3**
    Plausible function ($$Pl$$)
  ② The composition rules of multiple evidence in D-S evidence theory
    If multiple evidences could be combined by $m_{1} ,m_{2} ,m_{3} \ldots ,m_{n}$, they could be integrated into a new basic probability assignment by orthogonal sum operation.
    **Definition 4**
    For $\forall A \subseteq \Omega$, the D-S combination rule for identifying all countable mass functions $m_{1} ,m_{2} ,m_{3} \ldots ,m_{n}$ on the framework was as follows:
    4 $$m_{1} (A_{1} ) \oplus m_{2} (A_{2} ) \oplus \cdots \oplus m(A_{n} ) = \frac{1}{K}\sum {A_{1} \cap } A_{2} \cap \ldots \cap A_{n} =_{A} m_{1} (A_{1} ) * m_{2} (A_{2} ) * \cdots * m(A_{n} )$$
  ③ After all the evidence was integrated, new evidence was formed, and the basic probability assignment function, trust function, and plausible function of the new evidence were calculated according to the combination rule of D-S evidence theory.
  ④ We will judge the new evidence according to certain judgment criteria and get the conclusion we need.

Let $\Omega$ be the identification framework, and $m$ be the new basic probability assignment function after the integration of D-S evidence. The corresponding trust function and plausible function satisfied the definitions 2 and 3 in ①. The following two decision options were adopted:The decision based on trust function: according to the integration rule of D-S evidence theory, we got the trust function $Bel$ again, and the trust function $Bel$ was our decision result.

In order to find out the true value, the principle of "minimum point" was adopted. The main idea was that there was a set $A$ and the trust function was $Bel\left( A \right)$. if the set after removing a certain element in set $A$ was set to $B_{1}$, the trust function was $Bel\left( {B_{1} } \right)$, and $\left| {Bel\left( A \right) - Bel\left( {B_{1} } \right)} \right| < \alpha$, where $\alpha$ was the value set in advance, it was considered that the removed element did not affect the final result, and the process was repeated until all the remaining elements could not be removed, and the result was the final decision.

The decision based on the probability distribution function: let $A_{1}$, $A_{2}$ belonged to the set $\Omega$ and satisfied the following conditions:

5 $$\left\{ \begin{gathered} m(A_{1} ) = \max \left\{ {\left. {m(A_{i} ),A_{{\text{i}}} \subset \Omega } \right\}} \right. \hfill \\ m(A_{2} ) = \max \left\{ {\left. {m(A_{j} ),A_{i} \ne A_{j} } \right\}} \right. \hfill \\ \end{gathered} \right.$$

When there was: $$\left\{ \begin{aligned} & m(A_{1} ) - m(A_{2} ) > \alpha_{1} \hfill \\ & m(\Omega ) < \alpha_{2} \hfill \\ & m(A_{1} ) > m(\Omega ) \hfill \\ \end{aligned} \right.$$, then $A_{1}$ was the final decision, where $\alpha_{1}$, $\alpha_{2}$ was the preset value.

The D-S evidence theory has the capability to assimilate all relevant evidence that impacts the outcomes, thereby generating new conclusive evidence. This could be effectively employed in the multi-parameter early warning system for coal spontaneous combustion. This study proposed an optimal fusion procedure for D-S evidence, aiming to address the issue of significant conflict between evidence and the synthesised outcome, which deviated from common sense. The algorithm incorporated weight assignment as a key component. Despite the presence of conflicting evidence, it was still possible to achieve an optimal outcome through the process of integration. The synthesis formula could be expressed as follows:

6 $$m(A) =_{A} m_{1} (A_{1} ) * m_{2} (B_{j} ) * m_{3} (C_{l} ) + K * \frac{1}{n}\sum {{}_{l \le i \le n}m_{i} (A)}$$

In the formula: $K =_{\phi } m_{1} (A_{1} ) * m_{2} (B_{j} ) * m_{3} (C_{l} ) * \cdots$; $n$—the total number of evidence.

**Figure 5:**
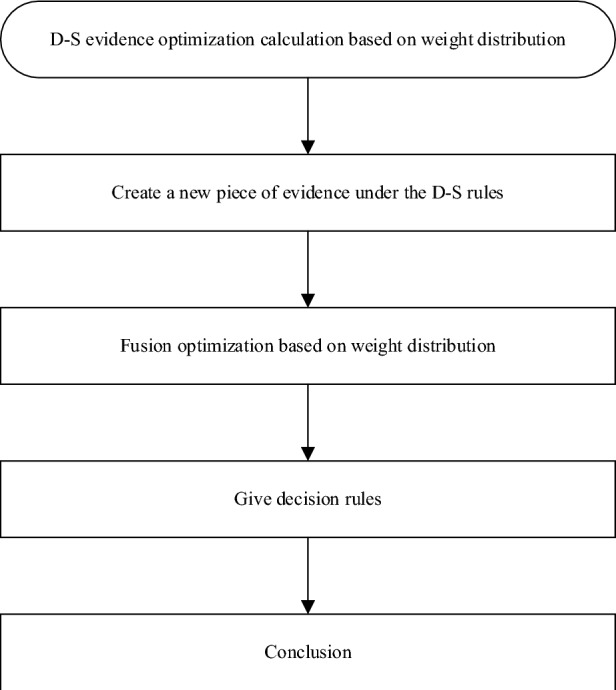
Fusion process of D-S evidence theory based on weight allocation.

In summary, the fusion process of D-S evidence theory based on weight allocation was shown in Fig. 5.

### Early warning level division

After the characteristic temperature, 100Δ(CO)/ΔO_2_ (%), C_3_H_8_/CH_4_, C_3_H_8_/C_2_H_6_, C_2_H_4_/C_2_H_6_, and C_2_H_2_ were fused according to the steps in Fig. 5, the goaf fire early warning index system was obtained, as shown in Table 2.

**Table 2:** Early warning index system of goaf fire in coal mine.

Development stage	Warning level	Temperature range	Early warning indicators	Index gases characteristics
Latent stage	Green warning	< 31.7 °C	100Δ(CO)/ΔO_2_(%): 0–0.02	O_2_ concentration slowly decreases and CO concentration increases slowly
Oxidation stage	Blue warning	31.7–54.8 °C	100Δ(CO)/ΔO_2_(%): 0.02–0.06	O_2_ concentration continues to decrease slowly and CO concentration rises faster
Critical stage	Yellow warning	54.8–153.7 °C	100Δ(CO)/ΔO_2_(%): 0.06–0.55 C_3_H_8_/CH_4_ > 0	C_3_H_8_/CH_4_ begins to rise rapidly
Pyrolysis stage	Orange warning	153.7–204.5 °C	100Δ(CO)/ΔO_2_(%): 0.55–1.55 C_3_H_8_/C_2_H_6_ > 0	C_3_H_8_/C_2_H_6_ begins to rise rapidly
Fission stage	Red warning	204.5–241.6 °C	100Δ(CO)/ΔO_2_(%) ≥ 1.55 C_2_H_4_/C_2_H_6_ > 0	O_2_ concentration decreases rapidly, CO concentration rises rapidly; C_2_H_4_/C_2_H_6_ begins to rise rapidly
Combustion stage	Black warning	> 241.6 °C	C_2_H_2_ > 0	C_2_H_2_ begins to appear

*Green warning*: At this time, the goaf fire is in the latent stage, all kinds of gas indicators are small, the temperature is low, only need to take goaf daily monitoring measures, do not need to take fire prevention and fire extinguishing measures.

*Blue warning*: At this time, it is in the oxidation stage, mainly chemical adsorption; the temperature rises slowly; and the coal body continues to store heat. We should strengthen the monitoring means of coal spontaneous combustion, immediately carry out relevant investigation and protection work, and do a good job of fire prevention and fire extinguishing measures.

*Yellow warning*: At this time, it is in the critical stage, the coal heating rate is fast, various gas indicators begin to appear, the goaf fire situation is analyzed, and effective measures are taken.

*Orange warning*: At this time, it is in the stage of pyrolysis, the fire in the goaf should be analyzed and effective measures should be taken to prevent and extinguish the fire by injecting fire-fighting materials into the goaf.

*Red warning*: At this time, it is in the fission stage, and there is a strong chemical reaction between coal and oxygen, so fire prevention and fire extinguishing materials should be injected into the goaf fire area in time, and the fire area should be closed at the same time.

*Black warning*: At this time, it is in the combustion stage, and the goaf has begun to fire, which may be accompanied by smoke and open fire. You should immediately close the fire area.

### Verification of early warning index system

**Figure 6:**
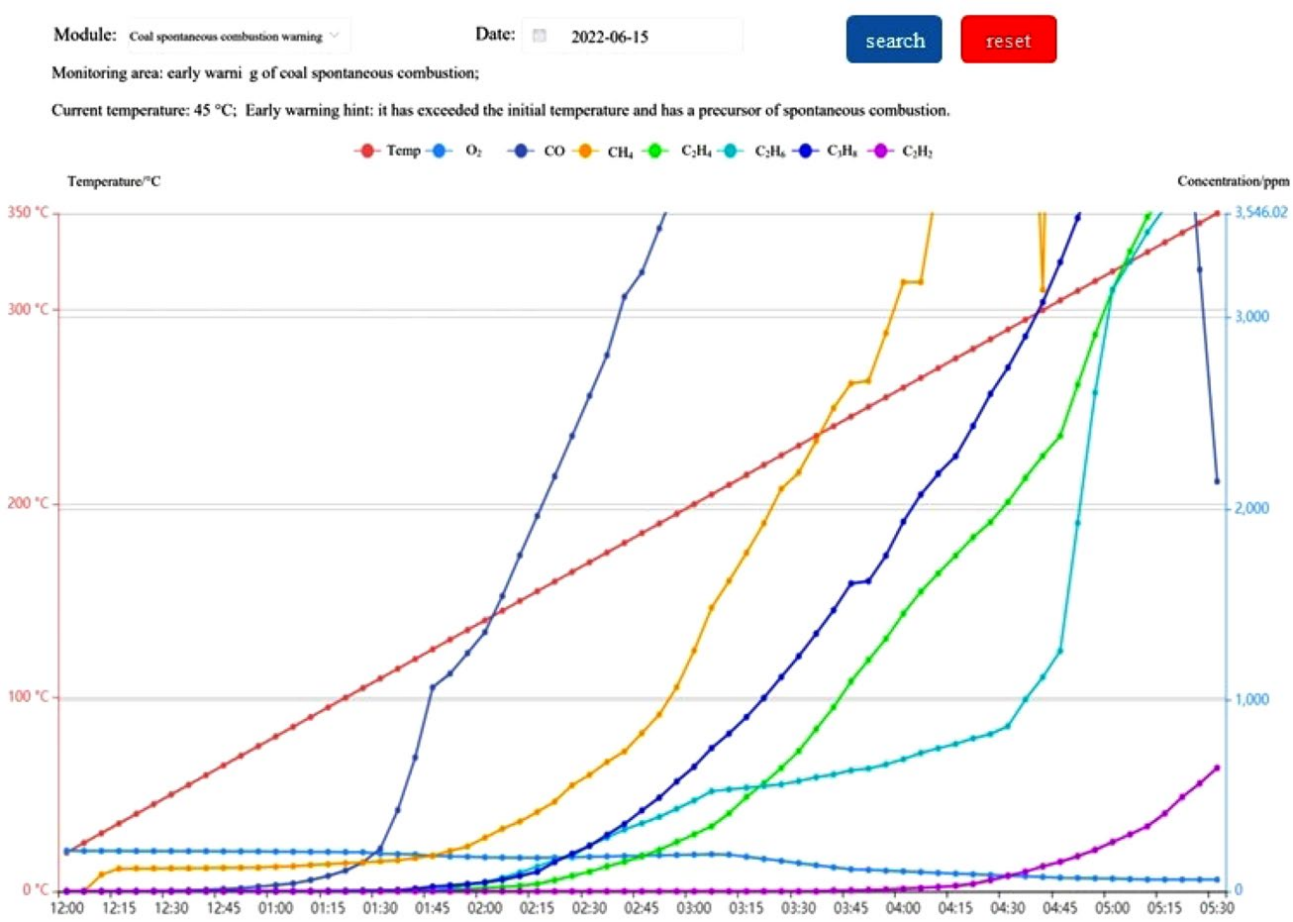
System monitoring interface.

A coal mine goaf fire multi-parameter fusion early warning system has been designed utilizing a page replacement algorithm and other approaches, based on the constructed multi-parameter fusion early warning index system. The system primarily consists of three functional modules: "real-time monitoring management, historical data management, and system management". The fundamental function is "real-time early warning and data processing," and the real-time monitoring interface of the system is depicted in Fig. 6.

**Figure 7:**
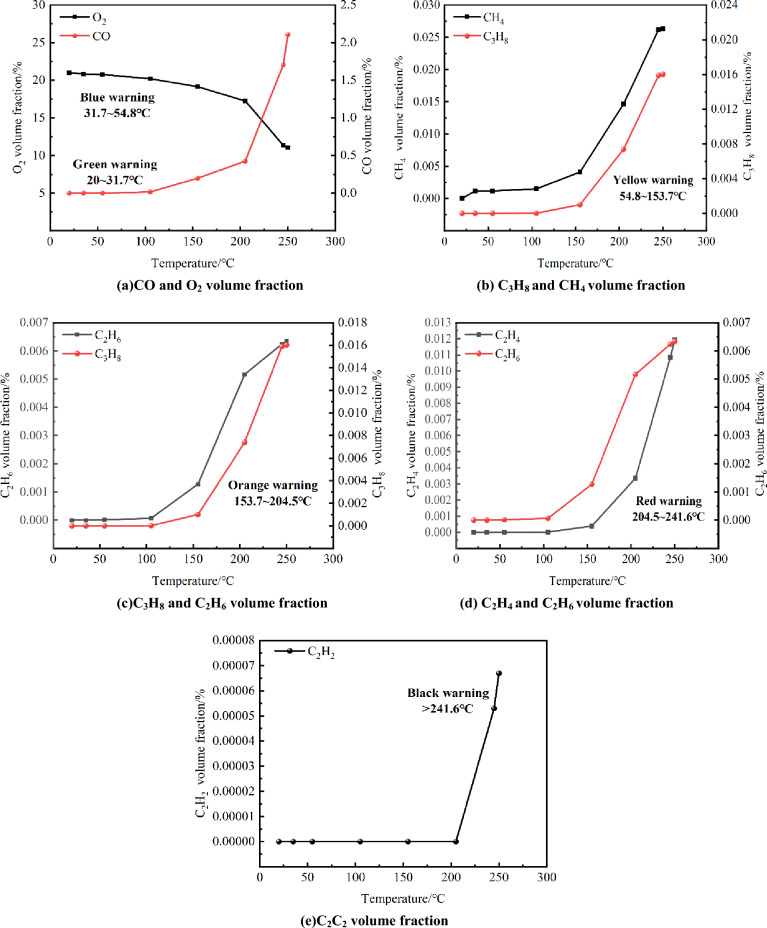
Data diagram of programmed heating experiment.

The reliability and accuracy of the early warning index system were verified by a programmed heating experiment, and the experimental data are shown in Fig. 7. When the experimental data were imported into the system, the results of system monitoring and analysis were as follows: There was no early warning before 35 °C, and the coal sample was in the latent stage; at 35 °C, the gas index value of 100Δ(CO)/ΔO_2_(%) was 0.024. And the software issued a blue warning prompt that the coal sample was in the stage of self-thermal oxidation. With the further rise in temperature, 100Δ(CO)/ΔO_2_(%) was 0.078 at 55 °C. And the software issued a yellow warning that the heat accumulation of coal samples entered the critical stage. Until C_3_H_8_ was detected at 105 °C, the ratio of 100Δ(CO)/ΔO_2_(%) was 0.530, and C_3_H_8_/CH_4_ was 0.011, which was still in the critical stage. When the temperature continued to rise to 155 °C, 100Δ(CO)/ΔO_2_(%) was 0.562, C_3_H_8_/C_2_H_6_ was 0.784, the software issued an orange warning prompt, the coal sample entered the pyrolysis stage. When the temperature reached 205 °C, 100Δ(CO)/ΔO_2_(%) was 1.566, C_2_H_4_/C_2_H_6_ was 0.650, the software issued a red warning warning that the coal sample entered the fission stage. When the temperature reached 245 °C, C_2_H_2_ was detected, the software issued a black warning, and the coal sample entered the combustion stage. In summary, the test results of the system were consistent with the actual situation in the process of coal spontaneous combustion. It was proved that the early warning index parameters and early warning grade division selected by the coal mine goaf fire early warning index system based on multi-parameter fusion have high reliability, and the prediction results have high accuracy.

## Conclusions

1. The coal spontaneous combustion process could be classified into six distinct stages, including the latent stage, oxidation stage, critical stage, pyrolysis stage, fission stage, and combustion stage. This classification was based on the analysis of thermogravimetric tests and programmed heating tests conducted on coal samples. The study concluded that temperatures of 31.7 °C, 54.8 °C, 153.7 °C, 204.5 °C, and 241.6 °C could serve as effective early warning temperature indicators for goaf fires. Additionally, a major gas index for early warning could be established using the ratio of 100 Δ(CO)/Δ(O_2_)%. Auxiliary gas indicators for early warning could include C_3_H_8_/CH_4_, C_3_H_8_/C_2_H_6_, C_2_H_4_/C_2_H_6_, and C_2_H_2_.
2. The multi-parameter fusion of goaf fire early warning temperature and gas index parameters was carried out by using D-S evidence theory, and then the D-S evidence was fused and optimized based on weight allocation. The goaf fire early warning index system was constructed as follows: green warning, T < 31.7 °C and 0 ≤ 100∆(CO)/∆O_2_(%) < 0.02; blue warning, 31.7 °C ≤ T < 54.8 °C and 0.02 ≤ 100∆(CO)/∆O_2_(%) < 0.06; yellow warning, 54.8 °C ≤ T < 153.7 °C, 0.06 ≤ 100Δ(CO)/ΔO_2_(%) < 0.55 and C_3_H_8_/CH_4_ > 0; orange warning, 153.7 °C ≤ T < 204.5 °C, 0.55 ≤ 100Δ(CO)/ΔO_2_(%) < 1.55 and C_3_H_8_/C_2_H_6_ > 0; red warning, 204.5 °C ≤ T < 241.6 °C, 100Δ(CO)/ΔO_2_(%) ≥ 1.55 and C_2_H_4_/C_2_H_6_ > 0; black warning, T ≥ 241.6 °C and C_2_H_2_ > 0.
3. The effectiveness of the coal mine goaf fire early warning index system, which utilizes multi-parameter fusion, has been confirmed through coal spontaneous combustion program heating experiments. The prediction results align with the actual progression of coal spontaneous combustion, thus validating the rationality of the selected early warning index parameters in this system. The early warning grade is dependable, and the early warning results exhibit exceptional precision and accuracy. The D-S evidence theory, utilizing weight allocation optimization, is employed to integrate the early warning index of goaf fire. This integration enhances the rationality and accuracy of the early warning index system, offering robust support for the development of a more comprehensive early warning system. Additionally, it provides an effective guarantee for precise prediction and forecasting of goaf fire.

## Data Availability

All data generated or analyzed during this study are included in this published article.
